# Association of ERCP Timing with Clinical Outcomes in Acute Cholangitis Secondary to Choledocholithiasis: A Retrospective Cohort Study

**DOI:** 10.3390/jcm15156000

**Published:** 2026-08-01

**Authors:** Tansu Ayyıldızoğlu, İbrahim Gören

**Affiliations:** 1 Department of Internal Medicine, Faculty of Medicine, Ondokuz Mayıs University, Samsun 55270, Türkiye; 2 Division of Gastroenterology, Department of Internal Medicine, Faculty of Medicine, Ondokuz Mayıs University, Samsun 55270, Türkiye; drigoren@hotmail.com

**Keywords:** acute cholangitis, ERCP timing, choledocholithiasis, mortality, qSOFA, Charlson Comorbidity Index

## Abstract

**Background/Objectives**: The optimal timing of endoscopic retrograde cholangiopancreatography (ERCP) in acute cholangitis remains controversial. This study evaluated the association between ERCP timing and clinical outcomes in patients with acute cholangitis secondary to choledocholithiasis. **Methods**: This retrospective cohort study included 271 consecutive patients treated between January 2020 and March 2025. Patients were categorized according to ERCP timing as ≤24 h, 24–72 h, and >72 h. Only patients with successful biliary cannulation and drainage during the index ERCP who did not require repeat ERCP were included. Disease severity was assessed using Tokyo 2018 severity grades, qSOFA scores, and Charlson Comorbidity Index (CCI). Hospital stay, intravenous antibiotic duration, complications, and 30-day mortality were analyzed. Corrected post-ERCP hospitalization duration was additionally calculated by excluding the admission-to-ERCP interval. **Results**: Age, CCI, and Tokyo severity grades were comparable among groups, whereas qSOFA scores differed significantly (*p* = 0.015). Admission neutrophil-to-lymphocyte ratio was highest in the ≤24 h group (*p* = 0.014). Overall complication rates were similar among groups (*p* = 0.136). Thirty-day mortality differed according to ERCP timing and was highest in patients undergoing ERCP within 24 h (16.7% vs. 5.6% vs. 6.6%, *p* = 0.020). Corrected post-ERCP hospital stay remained significantly longer in the >72 h group (*p* = 0.045). Multivariable analysis identified increasing age and CCI > 5 as independent predictors of mortality. After multivariable adjustment, ERCP performed between 24 and 72 h was associated with lower mortality than ERCP performed within 24 h, although residual confounding by indication cannot be excluded. Biliary stent placement was more common among older and more comorbid patients but was not associated with differences in mortality or other clinical outcomes. **Conclusions**: In patients with acute cholangitis secondary to choledocholithiasis, increasing age, greater comorbidity burden, and ERCP timing were independently associated with mortality. However, the higher mortality observed in patients undergoing ERCP within 24 h most likely reflected greater baseline physiological instability rather than a detrimental effect of early biliary drainage. Although delayed ERCP was associated with prolonged corrected post-procedural hospitalization, complication rates were similar across groups. The higher mortality observed in the ≤24 h group may reflect greater physiological instability at presentation rather than the effect of urgent ERCP itself. Similarly, no significant association was observed between biliary stent placement and clinical outcomes.

## 1. Introduction

Acute cholangitis is a potentially life-threatening gastrointestinal emergency that develops as a result of biliary obstruction and subsequent bacterial infection of the biliary tree. Despite substantial advances in critical care management, antimicrobial therapy, and endoscopic interventions, acute cholangitis remains associated with significant morbidity and mortality, particularly in elderly patients and those presenting with organ dysfunction or sepsis [1,2,3]. Early recognition and timely treatment are therefore essential to prevent progression to septic shock and multiple organ failure [4,5,6].

Biliary decompression represents the cornerstone of treatment in acute cholangitis [1]. Although percutaneous and surgical drainage techniques remain available in selected cases, endoscopic retrograde cholangiopancreatography (ERCP) has become the preferred method of biliary drainage because of its high technical success rate and favorable safety profile. Current guidelines uniformly recommend early biliary drainage in patients with acute cholangitis; however, the optimal timing of ERCP remains a matter of ongoing debate [4,7]. While the Tokyo Guidelines 2018 emphasize urgent or early drainage according to disease severity, they do not clearly define a universal time threshold applicable to all patients [8]. Similarly, international societies differ in their recommendations regarding whether ERCP should be performed within 12, 24, 48, or 72 h after presentation [9,10].

Several retrospective studies and recent meta-analyses have demonstrated an association between earlier ERCP and improved clinical outcomes, including reduced mortality and shorter hospital stay. Nevertheless, the available evidence remains largely observational and is characterized by substantial heterogeneity regarding patient populations, severity grading systems, etiologies of biliary obstruction, and definitions of early intervention [4,5]. Furthermore, many previous studies included patients with malignant biliary obstruction, benign strictures, stent dysfunction, and other causes of cholangitis, making it difficult to determine the specific impact of ERCP timing in patients with choledocholithiasis-related cholangitis [11,12].

An additional challenge in interpreting the existing literature is the potential influence of disease severity on treatment allocation. In routine clinical practice, patients with more severe cholangitis are more likely to undergo urgent ERCP, which may confound comparisons between early and delayed interventions. Consequently, the independent effects of ERCP timing may be difficult to distinguish from the effects of baseline disease severity, organ dysfunction, and comorbidity burden [13,14]. The incorporation of contemporary severity assessment tools, including the Tokyo 2018 severity grading system, qSOFA score, and Charlson Comorbidity Index, may provide a more comprehensive evaluation of factors influencing clinical outcomes [15,16].

Therefore, the present study aimed to evaluate the association between ERCP timing and clinical outcomes in patients hospitalized with acute cholangitis secondary to choledocholithiasis. In addition to conventional outcomes such as mortality, complications, hospital stay, and antibiotic utilization, we assessed baseline disease severity using Tokyo 2018 grades, qSOFA scores, and comorbidity burden. Furthermore, to minimize the confounding effect of pre-procedural waiting time, patient-level corrected post-ERCP hospitalization was analyzed. We hypothesized that the relationship between ERCP timing and clinical outcomes may be influenced not only by procedural timing itself but also by underlying disease severity and patient-related risk factors.

## 2. Materials and Methods

### 2.1. Study Design and Patient Population

As this was a retrospective chart review, ethical approval for retrospective analysis of the study data was obtained from the Ondokuz Mayıs University Clinical Research Ethics Committee (OMUKAEK No. 2025/571; Application No. 2025000571-1; Approval date: 24 November 2025).

The initial screening population was identified through the hospital information system by retrieving all admissions assigned an International Classification of Diseases (ICD) diagnostic code for acute cholangitis during the study period. Because this code-based screening strategy was designed to maximize sensitivity, it generated a broad pool of potentially relevant records. Each record was subsequently reviewed in detail to verify the diagnosis of acute cholangitis, determine its underlying etiology, confirm whether ERCP had been performed, and assess eligibility according to the predefined inclusion and exclusion criteria. A substantial proportion of the initially identified records represented miscoded or unconfirmed diagnoses, alternative biliary conditions, cases managed without ERCP, or records with insufficient clinical or follow-up information to confirm study eligibility.

Adult patients (≥18 years) who fulfilled the Tokyo Guidelines 2018 diagnostic criteria for acute cholangitis and had imaging-confirmed choledocholithiasis were eligible for inclusion. The diagnosis of choledocholithiasis was established by abdominal ultrasonography (US) and/or magnetic resonance cholangiopancreatography (MRCP).

Inclusion criteria were: (1) acute cholangitis secondary to choledocholithiasis confirmed by clinical, laboratory, and imaging findings; (2) performance of ERCP during the index hospitalization; (3) successful biliary cannulation during the first ERCP session; and (4) complete clinical and laboratory records available for review.

Exclusion criteria included age below 18 years, uncertain diagnosis of acute cholangitis, hospitalization for reasons other than acute cholangitis, incomplete clinical or laboratory data, recent chemotherapy or radiotherapy, acute myeloid or lymphocytic leukemia, malignant biliary obstruction, benign or malignant biliary strictures, primary sclerosing cholangitis, stent dysfunction, or acute cholangitis secondary to causes other than choledocholithiasis. Patients requiring a repeat ERCP during the same hospitalization for any reason were also excluded.

**Figure 1:**
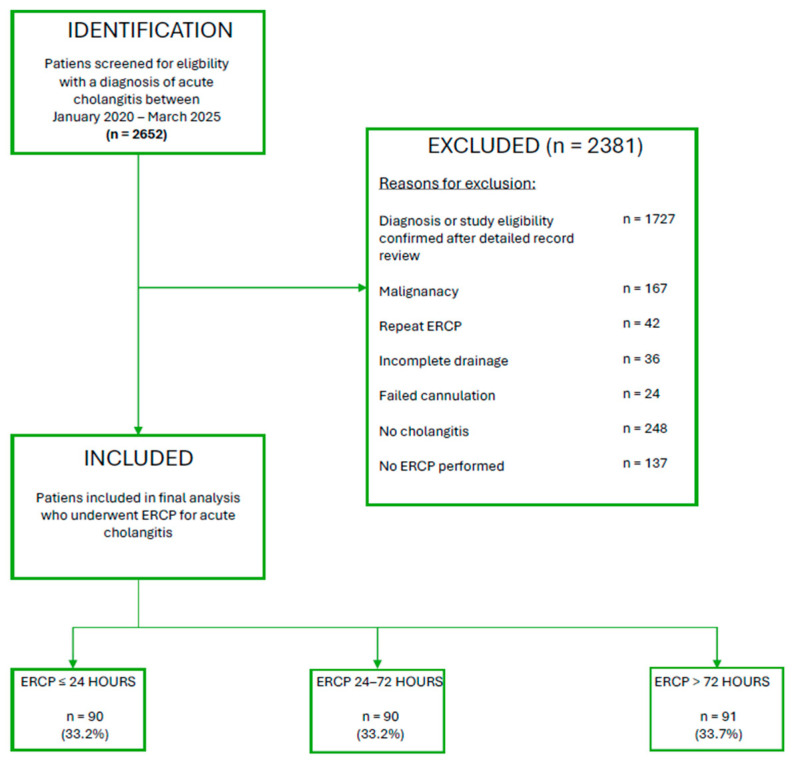
Flow diagram of patient selection and study inclusion.

A flow diagram illustrating patient screening, eligibility assessment, reasons for exclusion, and the final study cohort is presented in Figure 1.

### 2.2. ERCP Procedure and Medical Management

All ERCP procedures were performed by one of five endoscopists, each with experience exceeding 1000 ERCP procedures. A therapeutic duodenoscope system (Fujifilm ED-580XT or equivalent high-definition therapeutic duodenoscope; Fujifilm Corporation, Tokyo, Japan) was used in all procedures.

Only patients with successful common bile duct cannulation, biliary sphincterotomy, and complete extraction of bile duct stones and/or biliary sludge during the initial procedure were included in the study. Successful biliary drainage was achieved in all patients. In accordance with contemporary guideline recommendations, the primary therapeutic goal was adequate biliary decompression rather than routine stent placement. Therefore, temporary plastic biliary stents were placed selectively in patients in whom complete and sustained biliary drainage was considered potentially uncertain, including those with residual stone concerns, a perceived risk of early biliary obstruction, or at the discretion of the endoscopist based on intra-procedural findings. When stenting was required, plastic double pigtail biliary drainage catheters (Micro-Tech (Nanjing) Co., Ltd., Nanjing, China) with diameters ranging from 8.5 to 10 Fr and appropriate lengths according to the biliary anatomy were used. In patients with complete stone clearance and satisfactory bile flow, additional biliary stenting was not routinely performed [8,9,17].

Intravenous antibiotic therapy was initiated as early as possible upon admission in all patients. Antibiotic selection was individualized according to the patient’s clinical condition, comorbidities, drug allergy history, and local microbiological considerations, in consultation with an infectious diseases specialist. As specific antibiotic regimens were not systematically recorded, analyses according to antibiotic class or spectrum could not be performed.

### 2.3. Data Collection and Study Variables

Electronic medical records, laboratory results, endoscopy reports, intensive care unit records, and discharge summaries were retrospectively reviewed.

The following variables were collected: age, sex, ERCP timing, Charlson Comorbidity Index (CCI), qSOFA score, Tokyo 2018 severity grade, white blood cell count (WBC), neutrophil-to-lymphocyte ratio (NLR), procedure-related complications, intensive care unit admission, length of hospital stay, duration of intravenous antibiotic treatment, and 30-day mortality.

Clinical adverse outcomes included post-ERCP pancreatitis, persistent severe sepsis after ERCP, and procedure-related bleeding. Persistent severe sepsis after ERCP was defined as persistent systemic inflammatory response accompanied by hypotension requiring vasopressor support despite successful biliary drainage, adequate fluid resuscitation, and appropriate antimicrobial therapy during the index hospitalization. Cases were identified through retrospective review of the medical records, intensive care unit records, and discharge summaries during the index hospitalization. All patients were followed for at least 30 days after admission.

Thirty-day mortality was defined as all-cause mortality occurring within 30 days after hospital admission. Mortality status was verified for each patient using the Turkish Central Civil Registration System (MERNİS), which contains nationwide mortality records. In the original study database, the “exitus” variable was a binary indicator of vital status (alive/deceased), whereas the “30-day mortality” variable recorded the exact day of death after hospital admission for deceased patients, while survivors were coded as zero. Thus, the two variables were complementary rather than alternative mortality endpoints. For statistical analyses, the latter variable was converted into a binary outcome (death within 30 days: yes/no) and used consistently as the primary mortality endpoint throughout the study.

### 2.4. ERCP Timing Groups and Study Endpoints

Patients were categorized according to the interval between hospital admission and ERCP as follows:

* ERCP performed within 24 h (≤24 h);
* ERCP performed between 24 and 72 h;
* ERCP performed after 72 h (>72 h).

To account for differences in baseline comorbidity burden, the Charlson Comorbidity Index was calculated for all patients. In addition, qSOFA scores and Tokyo 2018 severity grades were recorded and compared among the three timing groups.

The primary endpoints were length of hospital stay, duration of intravenous antibiotic treatment, and 30-day mortality. Secondary endpoints included post-ERCP pancreatitis, persistent severe sepsis after ERCP, procedure-related bleeding, and intensive care unit admission.

Because total hospital stay inherently includes the pre-procedural waiting period, an additional patient-level corrected analysis was performed. For each patient, the admission-to-ERCP interval was recorded in hours, converted to days, and subtracted from the total hospital stay. Consequently, corrected post-ERCP hospital stay was calculated and analyzed.

This study was conducted and reported in accordance with the Strengthening the Reporting of Observational Studies in Epidemiology (STROBE) Statement.

### 2.5. Statistical Analysis

Statistical analyses were performed using IBM SPSS Statistics for Windows, Version 29.0 (IBM Corp., Armonk, NY, USA). Distribution of continuous variables was assessed using the Shapiro–Wilk test. Non-normally distributed variables were expressed as median and interquartile range (IQR) and compared using the Kruskal–Wallis test. When significant differences were detected, pairwise comparisons were performed using Dunn–Bonferroni correction. Categorical variables were expressed as frequencies and percentages and compared using Pearson’s chi-square test or Fisher’s exact test, as appropriate.

Multivariable logistic regression analysis was performed to identify independent predictors of 30-day mortality. Age was entered into the model as a 5-year increment. The Charlson Comorbidity Index was dichotomized as >5 versus ≤5. qSOFA was categorized as ≥2 versus <2. Tokyo Grade III patients were compared with Tokyo Grade II patients. ERCP performed within 24 h served as the reference category. Variables included in the multivariable model were prespecified a priori based on their established clinical relevance in acute cholangitis rather than solely on statistical significance. Although the number of mortality events was limited, qSOFA score and Tokyo 2018 severity grade were retained in the model because they represent established measures of disease severity and constituted key variables of interest in the present study. A two-sided *p*-value < 0.05 was considered statistically significant for all analyses.

## 3. Results

Baseline demographic and clinical characteristics are presented in Table 1. Age, Charlson Comorbidity Index (CCI), and Tokyo 2018 severity grades were comparable among the three ERCP timing groups. In contrast, sex distribution and qSOFA scores differed significantly between groups (*p* = 0.021 and *p* = 0.015, respectively). Grade III cholangitis constituted the majority of cases in all groups, accounting for more than three-quarters of the study population.

Laboratory findings according to ERCP timing are summarized in Table 2. Admission white blood cell (WBC) counts did not differ significantly among groups (*p* = 0.119). Similarly, WBC measurements obtained 24 and 48 h after ERCP were comparable across groups (*p* = 0.807 and *p* = 0.408, respectively). Admission neutrophil-to-lymphocyte ratio (NLR) differed significantly according to ERCP timing (*p* = 0.014). Post hoc analysis demonstrated that this difference was primarily attributable to lower admission NLR values in patients undergoing ERCP after 72 h compared with those treated within the first 24 h. No significant differences were observed in NLR values measured 24 or 48 h after ERCP.

Clinical outcomes are shown in Table 3. During hospitalization, 77 patients experienced at least one clinical adverse outcome. Persistent severe sepsis after ERCP was the most common adverse clinical outcome, occurring in 38 patients (14.0%), followed by post-ERCP pancreatitis in 24 patients (8.9%) and procedure-related bleeding in 10 patients (3.7%). The remaining adverse clinical outcomes consisted of infrequent isolated events, including anaphylaxis, perforation, lithotripter impaction, post-procedural psychosis, and chest pain requiring coronary angiography, each occurring only once and therefore not analyzed as separate outcome categories. Overall clinical adverse outcome rates were similar among ERCP timing groups (*p* = 0.136). Likewise, no significant differences were observed in the incidence of post-ERCP pancreatitis, persistent severe sepsis after ERCP, bleeding, ICU admission, or ICU length of stay among the ERCP timing groups. Thirty-day mortality, however, differed significantly according to ERCP timing (*p* = 0.020), with the highest mortality rate observed in patients who underwent ERCP within the first 24 h.

Persistent severe sepsis after ERCP was defined as persistent hypotension requiring vasopressor support despite successful biliary drainage, adequate fluid resuscitation, and appropriate antimicrobial therapy.

Corrected post-ERCP hospitalization outcomes are presented in Table 4. The median admission-to-ERCP interval was 24 (24–24) h in the ≤24 h group, 48 (36–60) h in the 24–72 h group, and 84 (72–96) h in the >72 h group (*p* < 0.001). After adjustment for the admission-to-ERCP interval, corrected post-ERCP hospital stay remained significantly different among the three ERCP timing groups (*p* = 0.045).

Independent predictors of 30-day mortality are presented in Table 5. Increasing age and higher comorbidity burden were independently associated with mortality. Each 5-year increase in age was associated with a 39% increase in the odds of death (OR 1.39, 95% CI 1.11–1.75; *p* = 0.005), while patients with a Charlson Comorbidity Index greater than 5 had more than fourfold higher odds of mortality than those with a score of 5 or less (OR 4.19, 95% CI 1.59–11.02; *p* = 0.004). Neither qSOFA ≥ 2 nor Tokyo Grade III remained independently associated with mortality in the multivariable model. Compared with ERCP performed within 24 h, ERCP performed between 24 and 72 h was associated with lower mortality (OR 0.24, 95% CI 0.08–0.74; *p* = 0.013), whereas ERCP performed after 72 h was not independently associated with mortality (OR 0.38, 95% CI 0.13–1.11; *p* = 0.077).

An additional analysis was performed according to biliary stent placement (Table 6). Patients who underwent biliary stent placement were older and had a higher Charlson Comorbidity Index than those who did not receive a stent. However, no significant differences were observed between the groups with respect to qSOFA scores, Tokyo 2018 severity grades, laboratory parameters, overall complication rates, post-ERCP pancreatitis, cholangiosepsis, bleeding, intensive care unit stay, or 30-day mortality. Although total hospital stay was longer in patients who underwent stent placement, this group also had a significantly greater comorbidity burden. Importantly, biliary stent placement was similarly distributed across the ERCP timing groups, occurring in 67.8% of patients in the ≤24 h group, 67.8% of patients in the 24–72 h group, and 67.0% of patients in the >72 h group (*p* = 0.992), suggesting that differences in stent utilization were unlikely to have influenced the observed associations between ERCP timing and clinical outcomes.

## 4. Discussion

This study evaluated the association between ERCP timing and clinical outcomes in a real-world cohort of patients with acute cholangitis secondary to choledocholithiasis. The principal findings were that patients undergoing ERCP within the first 24 h had the highest 30-day mortality, ERCP performed after 72 h was associated with a longer corrected post-ERCP hospital stay, and increasing age and comorbidity burden emerged as independent predictors of mortality. In contrast, ERCP timing was not associated with complication rates, intensive care unit utilization, or corrected post-ERCP antibiotic duration.

When evaluating the impact of ERCP timing on clinical outcomes, it is important to consider potential confounding factors, particularly comorbidity burden and disease severity. In retrospective real-world studies, age, underlying comorbidities, and clinical status at presentation are well-recognized determinants of mortality, complication rates, and length of hospitalization [18,19]. To minimize potential bias related to baseline health status, comorbidity burden was assessed using the Charlson Comorbidity Index (CCI). The CCI is a widely validated tool that has been shown to predict mortality and adverse clinical outcomes across various patient populations [20]. In the present study, no significant differences in CCI scores were observed among the three ERCP timing groups. This finding suggests that the groups were comparable with respect to baseline comorbidity burden and that differences in outcomes are unlikely to be explained solely by disparities in underlying chronic disease status. Although sex distribution differed significantly among the ERCP timing groups, the clinical significance of this isolated baseline difference remains uncertain because no corresponding differences were observed in the major clinical outcomes evaluated in the present study.

In addition to comorbidity burden, disease severity was assessed using the Tokyo 2018 severity grading system and qSOFA scores [8,16]. No significant differences were observed in Tokyo 2018 severity grades among the three ERCP timing groups. This finding indicates a comparable distribution of formally classified cholangitis severity across groups, irrespective of ERCP timing. In contrast, qSOFA score distribution differed significantly according to ERCP timing. Higher qSOFA scores were more frequently observed among patients who underwent ERCP within the first 24 h. This finding may reflect a tendency in routine clinical practice to prioritize earlier biliary drainage in patients presenting with greater physiological derangement or a higher risk of organ dysfunction. Accordingly, baseline clinical status should be considered when interpreting the relationship between ERCP timing and mortality outcomes.

Another notable finding was the significant difference in admission NLR values among ERCP timing groups. Patients who underwent ERCP within the first 24 h had the highest admission NLR values, whereas the lowest values were observed in patients treated after 72 h. Previous studies have demonstrated that elevated NLR is associated with severe cholangitis, bacteremia, septic shock, and adverse clinical outcomes [21,22]. In the present study, the higher NLR values observed in the ≤24 h group may further support the notion that patients selected for urgent ERCP represented a clinically more severe subgroup. However, NLR values measured 24 and 48 h after ERCP did not differ significantly among groups, suggesting that the early inflammatory response following biliary drainage was comparable regardless of ERCP timing.

Compared with mortality and hospital length of stay, the relationship between ERCP timing and procedure-related complications has been less extensively investigated. Most previous studies and meta-analyses have primarily focused on mortality, organ failure, intensive care utilization, and hospitalization outcomes, whereas data regarding post-ERCP pancreatitis, bleeding, and cholangiosepsis remain limited [4,7,23]. In the present study, no significant differences were observed in overall complication rates or in the incidences of post-ERCP pancreatitis, persistent severe sepsis after ERCP, and procedure-related bleeding according to ERCP timing. These findings are consistent with the limited available evidence suggesting that the timing of biliary drainage may have a greater impact on overall clinical outcomes than on short-term procedure-related complications. Although delayed ERCP was associated with longer corrected post-procedural hospitalization, it was not accompanied by a higher frequency of major complications in our cohort.

The relationship between ERCP timing and mortality remains one of the most controversial aspects of acute cholangitis management. In the present study, the highest 30-day mortality rate was observed among patients who underwent ERCP within the first 24 h. At first glance, this finding appears to contrast with several observational studies and recent meta-analyses reporting lower mortality rates among patients undergoing earlier biliary drainage [3,5,6]. In a systematic review and meta-analysis including more than 84,000 patients, Du et al. reported that ERCP performed within 24, 48, and 72 h was associated with lower in-hospital mortality compared with delayed intervention [4]. Similarly, another recent meta-analysis suggested that ERCP performed within 48 h was associated with improved mortality outcomes and shorter hospital stay [24]. However, not all contemporary evidence supports the superiority of urgent ERCP. In a recent randomized controlled trial, Jagtap et al. demonstrated that, among patients with mild-to-moderate acute cholangitis, ERCP performed within 24 h was not associated with lower mortality or reduced organ failure compared with ERCP performed within 24–48 h, while procedure-related adverse events were more frequent in the urgent ERCP group [6]. Therefore, the existing literature remains heterogeneous regarding the clinical benefit of very early biliary drainage. However, the interpretation of these findings requires caution, particularly in retrospective real-world cohorts where treatment allocation is not randomized.

A potential explanation for the higher mortality observed in the ≤24 h group may be related to differences in baseline clinical status rather than the timing of ERCP itself. Although Tokyo 2018 severity grades were similarly distributed among the three groups, qSOFA scores differed significantly according to ERCP timing. Patients undergoing ERCP within the first 24 h more frequently had qSOFA scores ≥ 2. This observation may indicate that, in routine clinical practice, patients with a less favorable clinical presentation were more likely to undergo earlier biliary drainage. Consequently, the higher mortality observed in the ≤24 h group could partially reflect differences in physiological status at presentation rather than the effect of ERCP timing alone. In contrast, Tokyo 2018 severity grades were comparable among groups. This finding may suggest that formal severity classification was not the sole factor influencing procedural timing. Other clinical considerations, including bedside assessment and the overall clinical impression of the treating physician, may also have contributed to the decision-making process regarding the timing of ERCP. Accordingly, the present findings should be interpreted as demonstrating an association rather than a causal effect of ERCP timing on mortality. Because treatment allocation was based on clinical judgment rather than randomization, no causal inference regarding the optimal timing of ERCP can be drawn from the present study.

The results of the multivariable analysis further support the importance of patient-related factors in determining mortality. Increasing age and a Charlson Comorbidity Index greater than 5 emerged as independent predictors of 30-day mortality, whereas qSOFA ≥ 2 and Tokyo Grade III did not retain statistical significance after adjustment. This finding should be interpreted cautiously because Tokyo Grade III cholangitis accounted for more than three-quarters of the study cohort, resulting in limited variability across the comparison groups, and the regression model simultaneously adjusted for complementary measures of disease severity and patient vulnerability, including qSOFA score and Charlson Comorbidity Index. The association between advanced age and adverse outcomes has been consistently demonstrated in previous studies of acute cholangitis and likely reflects reduced physiological reserve, greater frailty, and a higher prevalence of coexisting diseases [25,26]. Similarly, the strong association observed between a high comorbidity burden and mortality underscores the substantial impact of chronic health status on outcomes following an acute biliary event [27,28]. Notably, patients with a Charlson Comorbidity Index greater than 5 had more than fourfold higher odds of mortality, highlighting that underlying comorbid conditions may be at least as important as procedural timing in determining prognosis. The lack of an independent association between Tokyo Grade III and 30-day mortality should be interpreted cautiously. First, Tokyo Grade III was highly prevalent across all three ERCP timing groups, resulting in limited variability for multivariable discrimination. Second, the regression model simultaneously included qSOFA score and Charlson Comorbidity Index, both of which capture complementary aspects of physiological instability and underlying health status. Finally, because only patients who achieved successful biliary drainage during the index ERCP were included, patients with failed cannulation or more complex clinical courses were excluded. Together with the limited number of mortality events, these factors may have attenuated the independent prognostic contribution of Tokyo Grade III in the adjusted model.

An additional strength of the present study was the evaluation of corrected post-ERCP outcomes. Most previous studies assessing the impact of ERCP timing have used total hospital length of stay as an outcome measure [1,4,6,12,13,28]. However, total hospitalization inherently includes the pre-procedural waiting period and may therefore overestimate the effect of delayed intervention on recovery. To address this issue, we calculated corrected post-ERCP hospital stay by excluding the admission-to-ERCP interval on a patient-level basis. The pre-procedural period in acute cholangitis is not merely a passive waiting interval [29]. During this time, patients typically receive intravenous antibiotics, fluid resuscitation, hemodynamic optimization, and supportive care, all of which may influence subsequent clinical outcomes [2,30]. In selected patients, correction of coagulation abnormalities, optimization of comorbid conditions, or anesthetic evaluation may also be required before ERCP [31]. Therefore, a proportion of the clinical improvement observed after biliary drainage may already begin before the procedure itself [32]. This should be considered when interpreting studies that use total hospital stay as a surrogate marker of recovery. Using this approach, patients who underwent ERCP after 72 h continued to demonstrate a longer post-procedural hospital stay compared with those treated between 24 and 72 h. In contrast, corrected post-ERCP antibiotic duration did not differ significantly among groups. These findings suggest that although delayed ERCP may be associated with prolonged recovery after biliary drainage, the differences reported in total hospitalization may not be entirely attributable to the post-procedural period alone. Consequently, part of the increase in overall hospital stay observed in delayed ERCP groups may reflect the pre-procedural optimization phase rather than a slower recovery following ERCP itself.

An additional exploratory analysis was performed to evaluate whether selective biliary stent placement influenced the observed clinical outcomes. Current guidelines emphasize that the principal therapeutic objective in acute cholangitis is adequate biliary decompression rather than routine biliary stenting [8,9,17]. Accordingly, all patients included in the present study achieved successful biliary drainage during the index ERCP procedure, whereas biliary stents were inserted selectively when sustained drainage was considered potentially uncertain based on procedural findings and endoscopist judgment. To assess the potential impact of this practice pattern, outcomes were compared between patients who underwent biliary stent placement and those who did not. Interestingly, patients receiving biliary stents were significantly older and had a greater comorbidity burden. Previous studies have reported that elderly patients with acute cholangitis may present with greater clinical vulnerability and a higher frequency of severe disease despite similar mortality and hospitalization outcomes compared with younger patients [33,34,35]. Therefore, it is plausible that endoscopists in our cohort were more inclined to place biliary stents in older and more comorbid patients because of concerns regarding recurrent biliary obstruction or inadequate drainage following ERCP. Nevertheless, despite these less favorable baseline characteristics, no significant differences were observed in 30-day mortality, overall complication rates, post-ERCP pancreatitis, cholangiosepsis, bleeding, or intensive care unit utilization between patients with and without stent placement. Furthermore, stent placement was similarly distributed across the ERCP timing groups. Taken together, these findings support current guideline recommendations suggesting that successful biliary drainage itself may be more important than routine stent placement once adequate ductal clearance and bile flow have been achieved [9]. However, because retained or forgotten biliary stents may lead to clinically significant complications, particularly in elderly and medically complex patients, careful follow-up remains essential whenever temporary biliary stents are used [36].

An additional consideration is the relatively high proportion of patients with Tokyo Grade III cholangitis who underwent ERCP more than 72 h after admission. Although current guidelines generally recommend urgent biliary drainage in patients with severe cholangitis, real-world clinical practice may differ because procedural timing is influenced by multiple factors beyond disease severity alone. Delays may occur because of the need for hemodynamic stabilization, correction of coagulopathy, optimization of significant comorbidities, anesthetic assessment, temporary limitations in endoscopy unit availability, or referral from outside institutions. These factors were not systematically collected or documented as study variables in the present study and are therefore presented only as plausible explanations based on routine clinical practice rather than findings directly derived from our dataset. Therefore, ERCP timing in routine clinical practice does not always strictly follow guideline-recommended time thresholds. This should be considered when interpreting the external generalizability of the present findings [8,10].

Several limitations of the present study should be acknowledged. First, owing to its retrospective design, the influence of selection bias and unmeasured confounding factors cannot be completely eliminated. In particular, ERCP timing was not determined according to a predefined protocol but rather according to the patient’s clinical condition and the judgment of the treating endoscopist. Although no significant differences were observed among groups with respect to Tokyo 2018 severity grades or Charlson Comorbidity Index scores, patients undergoing ERCP within the first 24 h had significantly higher qSOFA scores. This finding may indicate that these patients presented with greater physiological instability at admission and could have contributed to the higher mortality observed in the early ERCP group. Nevertheless, most previous studies evaluating ERCP timing have relied primarily on Tokyo severity grading alone, whereas the combined assessment of qSOFA and comorbidity burden has been less frequently reported. Therefore, despite its retrospective nature, the present study provides an additional perspective by incorporating multiple dimensions of disease severity into the analysis. In addition, the study population was identified through an initial screening of all hospital admissions assigned an ICD diagnosis code for acute cholangitis, followed by detailed chart review to confirm study eligibility. Because this screening strategy prioritized sensitivity, many initially identified records were subsequently excluded owing to miscoded or unconfirmed diagnoses, alternative etiologies, patients who did not undergo ERCP as part of their clinical management, or insufficient documentation to confirm study eligibility. As detailed baseline information was collected only for the final eligible cohort, comparisons between included and excluded patients could not be performed. This should be considered when interpreting the potential for selection bias. Second, patients undergoing delayed ERCP received varying durations of pre-procedural medical treatment, including intravenous antibiotics, fluid resuscitation, hemodynamic support, and other supportive interventions. As these measures may influence clinical outcomes independently of biliary drainage, the isolated effect of ERCP timing cannot be fully determined. Although a patient-level corrected analysis was performed for hospital length of stay, the impact of pre-procedural optimization cannot be completely excluded. In addition, exclusion of patients with physiologically implausible negative corrected values may have introduced a small degree of selection bias, although only a limited proportion of patients was affected. Third, antibiotic therapy was individualized according to the patient’s clinical status, allergy profile, prior antibiotic exposure, and recommendations from infectious diseases specialists. However, the specific antibiotic regimens and antimicrobial spectrum were not systematically recorded. Consequently, the potential influence of different antibiotic strategies on clinical outcomes could not be evaluated. Fourth, only patients with successful common bile duct cannulation during the initial ERCP session and those who did not require a repeat ERCP during the same hospitalization were included. This approach was chosen to create a more homogeneous study population and to minimize the confounding effect of repeated interventions on clinical outcomes. However, excluding patients with failed initial cannulation or those requiring repeat ERCP may have preferentially removed technically more complex and clinically unstable cases from the analysis, thereby introducing selection bias. As a result, the present study may have underestimated the frequency of adverse clinical outcomes that would be observed in an unselected real-world ERCP population. Accordingly, caution should be exercised when extrapolating these findings to patients with more challenging biliary anatomy or complex endoscopic interventions. Finally, because only 26 deaths occurred during follow-up, the events-per-variable ratio in the multivariable regression model was relatively low. Although the variables included in the model were prespecified based on their established clinical relevance, the limited number of events may have affected the stability of the regression estimates, particularly for variables with wider confidence intervals. Therefore, the multivariable findings should be interpreted with appropriate caution.

## 5. Conclusions

In this real-world cohort of patients with acute cholangitis secondary to choledocholithiasis, ERCP timing was associated with several clinically relevant outcomes. Patients undergoing ERCP within the first 24 h exhibited the highest 30-day mortality, whereas ERCP performed after 72 h was associated with a longer corrected post-ERCP hospital stay. However, increasing age and comorbidity burden emerged as independent predictors of mortality. After multivariable adjustment, ERCP performed between 24 and 72 h was associated with lower mortality than ERCP performed within 24 h. However, this adjusted association should be interpreted cautiously because residual confounding by indication cannot be excluded. The higher mortality observed in the early ERCP group may reflect greater physiological instability at presentation rather than an adverse effect of urgent biliary drainage itself. This interpretation is further supported by the significantly higher qSOFA scores and admission NLR values observed in this group. Accordingly, these findings should be interpreted as associations observed in routine clinical practice rather than evidence of a causal relationship between ERCP timing and mortality. Furthermore, the use of corrected post-ERCP outcomes suggests that part of the prolonged hospitalization observed in delayed ERCP groups may be attributable to the pre-procedural period rather than the recovery phase alone. Similarly, although biliary stent placement was more common among older and more comorbid patients, no significant association was observed between stent placement and clinical outcomes. Prospective studies incorporating standardized severity assessment and treatment protocols are needed to better determine whether ERCP timing independently influences clinical outcomes in acute cholangitis.

No identifiable personal data, images, or videos of individual participants are included in this manuscript.

## Figures and Tables

**Table 1:** Baseline demographic and clinical characteristics.

Variable	≤24 h (*n* = 90)	24–72 h (*n* = 90)	>72 h (*n* = 91)	*p*-Value
Age, years	71.0 (61.0–80.8)	70.5 (63.0–83.0)	68.0 (59.0–81.0)	0.313
Male sex, n (%)	49 (54.4)	52 (57.8)	35 (38.5)	0.021
CCI	5.0 (3.0–6.0)	5.0 (2.2–6.0)	5.0 (3.0–6.0)	0.674
qSOFA 0, n (%)	45 (50.0)	61 (67.8)	60 (65.9)	
qSOFA 1, n (%)	25 (27.8)	21 (23.3)	22 (24.2)	
qSOFA 2, n (%)	19 (21.1)	8 (8.9)	6 (6.6)	
qSOFA 3, n (%)	1 (1.1)	0 (0.0)	3 (3.3)	0.015
Tokyo Grade II, n (%)	16 (17.8)	18 (20.0)	21 (23.1)	
Tokyo Grade III, n (%)	74 (82.2)	72 (80.0)	70 (76.9)	0.673

Abbreviations: CCI, Charlson Comorbidity Index; qSOFA, quick Sequential Organ Failure Assessment; IQR, interquartile range. Continuous variables are presented as median (IQR); categorical variables are presented as n (%). *p* values for qSOFA score and Tokyo 2018 severity grade refer to comparisons of the overall distribution across categories.

**Table 2:** Laboratory parameters according to ERCP timing.

Variable	≤24 h	24–72 h	>72 h	*p*-Value
WBC at diagnosis	10,545 (7812–14,758)	10,200 (8072–14,945)	9480 (6990–13,300)	0.119
WBC 24 h after ERCP	7970 (6540–11,185)	8925 (6762–12,730)	8360 (6285–12,070)	0.807
WBC 48 h after ERCP	7675 (6408–9298)	8370 (6200–10,905)	8680 (6450–11,225)	0.408
NLR at diagnosis	10.26 (4.77–20.33)	8.06 (4.57–18.49)	6.07 (4.09–11.76)	0.014
NLR 24 h after ERCP	6.64 (4.30–9.93)	5.62 (3.68–8.61)	4.88 (3.62–8.04)	0.118
NLR 48 h after ERCP	4.92 (2.98–8.26)	4.04 (2.79–7.32)	4.34 (3.22–6.55)	0.378

Abbreviations: WBC, white blood cell count; NLR, neutrophil-to-lymphocyte ratio; ERCP, endoscopic retrograde cholangiopancreatography; IQR, interquartile range.

**Table 3:** Clinical outcomes according to ERCP timing.

Outcome	≤24 h	24–72 h	>72 h	*p*-Value
Any complication, n (%)	32 (35.6)	20 (22.2)	25 (27.5)	0.136
Post-ERCP pancreatitis, n (%)	9 (10.0)	6 (6.7)	9 (9.9)	0.670
Persistent severe sepsis after ERCP, n (%)	16 (17.8)	8 (8.9)	14 (15.4)	0.206
Bleeding, n (%)	5 (5.6)	2 (2.2)	3 (3.3)	0.480
30-day mortality, n (%)	15 (16.7)	5 (5.6)	6 (6.6)	0.020
ICU admission, n (%)	11 (12.2)	5 (5.6)	8 (8.8)	0.290
ICU length of stay, days	0.0 (0.0–0.0)	0.0 (0.0–0.0)	0.0 (0.0–0.0)	0.275

Abbreviations: ERCP, endoscopic retrograde cholangiopancreatography; ICU, intensive care unit.

**Table 4:** Patient-level corrected post-ERCP hospital stay.

Variable	≤24 h	24–72 h	>72 h	*p*-Value
Admission-to-ERCP interval, hours, median (IQR) [range]	24 (24–24) [12–24]	48 (36–60) [36–60]	84 (72–96) [72–108]	<0.001
Corrected post-ERCP hospital stay, days, median (IQR) (*n* = 267)	6.0 (4.0–10.4)	5.5 (3.5–9.0)	8.0 (4.5–14.3)	0.045

Abbreviations: ERCP, endoscopic retrograde cholangiopancreatography; IQR, interquartile range. Footnote: Corrected post-ERCP hospital stay was calculated on a patient-by-patient basis by subtracting the admission-to-ERCP interval (converted from hours to days) from the total hospital stay. Data are presented as median (IQR).

**Table 5:** Multivariable logistic regression analysis for 30-day mortality.

Variable	Adjusted OR	95% CI	*p*-Value
Age (per 5-year increase)	1.39	1.11–1.75	0.005
CCI > 5	4.19	1.59–11.02	0.004
qSOFA ≥ 2	1.91	0.63–5.79	0.251
Tokyo Grade III (vs. Grade II)	0.77	0.23–2.64	0.678
ERCP 24–72 h (vs. ≤24 h)	0.24	0.08–0.74	0.013
ERCP > 72 h (vs. ≤24 h)	0.38	0.13–1.11	0.077

Abbreviations: OR, odds ratio; CI, confidence interval; CCI, Charlson Comorbidity Index; qSOFA, quick Sequential Organ Failure Assessment; ERCP, endoscopic retrograde cholangiopancreatography. Footnote: Age was entered into the model as a 5-year increment. CCI was dichotomized as >5 versus ≤5. qSOFA was dichotomized as ≥2 versus <2. Tokyo Grade III was compared with Tokyo Grade II. The ≤24 h ERCP group served as the reference category.

**Table 6:** Baseline characteristics, laboratory findings, and clinical outcomes according to biliary stent placement.

Variable	Stent (−) (*n* = 88)	Stent (+) (*n* = 183)	*p*-Value
**Baseline characteristics**			
Age, years	66.5 (56.5–75.0)	73.0 (64.5–82.5)	0.001
Male sex, n (%)	42 (47.7)	94 (51.4)	0.572
CCI	4.0 (2.0–5.3)	5.0 (3.0–6.0)	0.002
qSOFA 0, n (%)	53 (60.2)	113 (61.7)	
qSOFA 1, n (%)	20 (22.7)	48 (26.2)	
qSOFA 2, n (%)	12 (13.6)	21 (11.5)	
qSOFA 3, n (%)	3 (3.4)	1 (0.5)	0.279
Tokyo Grade II, n (%)	15 (17.0)	40 (21.9)	
Tokyo Grade III, n (%)	73 (83.0)	143 (78.1)	0.447
**Laboratory findings**			
WBC at diagnosis	10,585 (8355–15,008)	9700 (7030–14,370)	0.078
WBC 24 h after ERCP	8305 (6500–10,983)	8540 (6355–12,160)	0.944
WBC 48 h after ERCP	7690 (6475–10,148)	8200 (6360–10,990)	0.610
NLR at diagnosis	8.64 (5.05–15.77)	7.92 (4.12–14.72)	0.223
NLR 24 h after ERCP	5.55 (4.26–8.13)	5.50 (3.58–9.38)	0.785
NLR 48 h after ERCP	4.23 (3.19–7.64)	4.78 (3.04–6.61)	0.893
**Clinical outcomes**			
Any complication, n (%)	27 (30.7)	50 (27.3)	0.568
Post-ERCP pancreatitis, n (%)	8 (9.1)	16 (8.7)	1.000
Persistent severe sepsis after ERCP, n (%)	13 (14.8)	25 (13.7)	0.852
Bleeding, n (%)	4 (4.5)	6 (3.3)	0.732
30-day mortality, n (%)	9 (10.2)	17 (9.3)	0.827
ICU length of stay, days	0.0 (0.0–0.0)	0.0 (0.0–0.0)	0.691
IV antibiotic duration, days	6.0 (4.0–11.0)	8.0 (5.0–12.0)	0.100
Hospital stay, days	7.0 (5.0–11.3)	9.0 (6.0–14.5)	0.010

Abbreviations: CCI, Charlson Comorbidity Index; qSOFA, quick Sequential Organ Failure Assessment; WBC, white blood cell count; NLR, neutrophil-to-lymphocyte ratio; ERCP, endoscopic retrograde cholangiopancreatography; ICU, intensive care unit. Continuous variables are presented as median (IQR), and categorical variables as n (%).

## Data Availability

The datasets generated and analyzed during the current study are not publicly available due to ethical restrictions and institutional policies regarding patient confidentiality. However, de-identified data may be made available from the corresponding author upon reasonable request and with appropriate ethical approvals.
